# Bioactivity of Fungal Endophytes as a Function of Endophyte Taxonomy and the Taxonomy and Distribution of Their Host Plants

**DOI:** 10.1371/journal.pone.0073192

**Published:** 2013-09-16

**Authors:** Sarah J. Higginbotham, A. Elizabeth Arnold, Alicia Ibañez, Carmenza Spadafora, Phyllis D. Coley, Thomas A. Kursar

**Affiliations:** 1 Smithsonian Tropical Research Institute, Panama, Republic of Panama; 2 School of Plant Sciences, University of Arizona, Tucson, Arizona, United States of America; 3 Department of Biology, University of Utah, Salt Lake City, Utah, United States of America; 4 Instituto de Investigaciones Científicas y Servicios de Alta Tecnología, Panama, Republic of Panama; University of California Riverside, United States of America

## Abstract

Fungal endophytes – fungi that grow within plant tissues without causing immediate signs of disease – are abundant and diverse producers of bioactive secondary metabolites. Endophytes associated with leaves of tropical plants are an especially exciting and relatively untapped source of novel compounds. However, one major challenge in drug discovery lies in developing strategies to efficiently recover highly bioactive strains. As part of a 15-year drug discovery project, foliar endophytes were isolated from 3198 plant samples (51 orders, 105 families and at least 232 genera of angiosperms and ferns) collected in nine geographically distinct regions of Panama. Extracts from culture supernatants of >2700 isolates were tested for bioactivity (*in vitro* percent inhibition of growth, % IG) against a human breast cancer cell line (MCF-7) and the causative agents of malaria, leishmaniasis, and Chagas' disease. Overall, 32.7% of endophyte isolates were highly active in at least one bioassay, including representatives of diverse fungal lineages, host lineages, and collection sites. Up to 17% of isolates tested per assay were highly active. Most bioactive strains were active in only one assay. Fungal lineages differed in the incidence and degree of bioactivity, as did fungi from particular plant taxa, and greater bioactivity was observed in endophytes isolated from plants in cloud forests vs. lowland forests. Our results suggest that using host taxonomy and forest type to tailor plant collections, and selecting endophytes from specific orders or families for cultivation, will markedly increase the efficiency and efficacy of discovering bioactive metabolites for particular pharmaceutical targets.

## Introduction

Poor nutrition, a lack of clean water and proper sanitation, global climate change, population aging, pollution, and the emergence of drug-resistant pathogens together contribute to the economic and human challenges of today's global disease burden [1] [2] [3] [4] [5]. As one of the leading causes of mortality worldwide, cancer accounted for 7.6 million deaths in 2008 (13% of all deaths in that year), and is expected to cause an estimated 13.1 million deaths annually by 2030. Neglected tropical diseases, including leishmaniasis and Chagas' disease, have a global disease burden of on par with that of cancer (11.4% of annual deaths), with malaria alone causing an estimated 655,000 deaths in 2008 (mostly of African children) [1] [2] [3]. Yet of all the drugs approved between 1975 and 2004, only 1.3% (21 of 1556) were developed specifically to treat neglected tropical diseases [4], and treatments for cancer remain elusive in many cases [5].

After peaking during the ‘Golden Age of Antibiotics’ in the first half of the 20^th^ century, the pharmaceutical industry's interest in natural products and natural product structures as a source of drug leads has gradually decreased [6]. However, drug discovery from natural products is far from exhausted [7] [8] [9] [10]. New methods for screening, new approaches for engineering novel products from natural scaffolds, and the emergence of new diseases argue for re-evaluation of drug discovery processes, especially with regard to natural products from under-explored sources [11]. As of 2005, approximately 22,000 bioactive secondary metabolites from microorganisms had been described in published works. About 8,600 (38%) of these are of fungal origin [10], highlighting the biochemical richness of this diverse clade of eukaryotes.

Endophytic fungi are microfungi that grow within plant tissues without causing immediate symptoms of disease [12]. Some provide benefits to their hosts including improved drought tolerance (e.g., [13]), protection against pathogens (e.g., [14]), enhanced growth (e.g., [15]), and defense against herbivory (e.g., [16] [17]). These features, combined with their immense diversity (e.g., [14]), led drug discovery scientists to consider endophytic fungi as sources of potentially interesting metabolites. Recent reviews report the characterization of 138 secondary metabolites from endophytic fungi before 2000 [18] with an additional 184 reported by 2006 [19]. These metabolites encompass a diverse range of structures including alkaloids [20], terpenoids [21], quinones [22], and peptides, xanthones and phenols [23]. Bioactivity has been observed against cancer cell lines [24] [25], pathogenic bacteria [26] [27] and fungi (e.g., [28]), and against eukaryotic parasites such as the causal agents of malaria, leishmaniasis and Chagas' disease (e.g., [29] [30]).

One major challenge in drug discovery based on endophytic fungi lies in developing efficient strategies to recover bioactive strains. Strobel and Daisy [31] suggested that areas of high biodiversity and with high numbers of endemic plant species may hold the most potential for endophytes with novel chemistry. Tropical forests are some of the most biodiverse ecosystems on earth and tropical leaves are ‘biodiversity hotspots’ in terms of the endophytes they harbor [32]. However, surveys of tropical endophytes often yield very large culture libraries (e.g., [31] [32]) for which broad screening can be inefficient and costly. Here we use the results of a long-term drug discovery project to explore how tailoring search criteria in tropical forests could significantly enhance the discovery rate of bioactive foliar endophytes.

Despite the enormous natural wealth of the world's tropical forests, an ever-widening disparity exists between countries with the financial resources to develop potential leads, and biodiversity-rich countries with underdeveloped or developing economies that may be unable to capitalize upon these important natural resources [33]. The International Collaborative Biodiversity Groups (ICBG; [34]) aim to link the infrastructure, training and experience of academic and industrial organizations in developed nations with the potential of the tropical countries that are guardians to extraordinary biological and biochemical richness. Specifically, ICBGs promote sustainable use of ecological wealth while providing training and infrastructure to local communities and maintaining a collaborative pipeline for drug development and other applications [35].

For the past 15 years the Panama ICBG has applied ecological insights to guide the search for novel, bioactive compounds from terrestrial sources [36]. Diverse plants have been collected from forests throughout Panama and their endophytic fungi have been isolated in culture. Crude extracts have been tested for bioactivity against a breast cancer cell line and the causal agents of malaria, leishmaniasis and Chagas' disease. Identification of these fungi using molecular analysis provides an opportunity to look for broad patterns in bioactivity not only at the genotype or strain level, but at higher taxonomic levels that may in turn assist in focusing future surveys. Specifically, our collection of tropical endophytes provides a basis for assessing (1) the prevalence, variation, and specificity of bioactivity in particular fungal lineages; (2) patterns of bioactivity among endophytes from particular plant taxa; and (3) patterns of bioactivity among endophytes from particular forest types (cloud forest vs. lowland forest).

Here, we highlight the prevalence and specific bioactivity of particular lineages of endophytes; reveal differences in bioactivity among endophytes from particular plant lineages; and show for the first time that endophytes obtained in cloud forests are more likely to be bioactive than those obtained in proximate lowland forests. Elucidating such patterns may help guide future drug discovery efforts from tropical endophytic fungi, making the most of limited resources and maximizing the chances of encountering novel and active biochemistry.

## Materials and Methods

Appropriate collection permits were obtained from Panama's Autoridad Nacional del Ambiente (ANAM) and healthy plant tissues were collected in national parks throughout Panama (Table 1). Leaves were stored in plastic bags and kept cool until processed, usually within 24 hours. Plants were identified using taxonomic keys and floras, as well as comparisons with collections in the herbaria of the University of Panama (PMA) and Missouri Botanical Garden (MO). Voucher specimens were deposited in PMA, MO, and at the herbarium of the Smithsonian Tropical Research Institute (SCZ).

**Table 1:** Forest type, location, approximate area of each forest reserve, and mean annual temperature and rainfall of collection sites; number of host plant families collected (minimum number of genera, minimum number of genotypes); number of endophytic fungi isolated and isolates sequenced (number of genotypes); and number of isolates (minimum number of genotype groups) tested in each bioassay.

								Endophytes		Fungal Isolates Tested in Bioassays			
Collection Site	Forest Type	Approx. Location	Forest Area (km2)	Mean Annual Temp (°C)	Mean Annual Precip (mm)	Host Plant Samples	Host Plant Families	Isolated	Sequenced	Malaria	Leish-maniasis	Chagas' Disease	Cancer
Altos de Campana National Park	Cloud	8°41”N /79° 55”W	42.9	24	2500	175	41 (35, 13)	204	21 (18)	131(7)	152 (9)	147 (9)	161 (11)
Barro Colorado Island	Lowland Humid	9°94”N /79° 47”W	112	28	2600	903	63 (115, 118)	903	512 (152)	644 (118)	724 (131)	733 (130)	703 (130)
Chagres National Park	Lowland Dry, Humid; Cloud	9°14”N /79° 22”W	1311.4	30	3100	480	21 (19, 13)	480	67 (44)	377 (35)	380 (35)	393 (36)	390 (36)
Coiba National Park and Buffer Zones	Lowland Humid	7°30”N /81° 51”W	523.7	26	3500	952	39 (66, 54)	1032	459 (143)	407 (72)	625 (123)	622 (114)	649 (122)
Fortuna Forest Reserve	Cloud	8°40”N /81° 21”W	206.5	20	5500	171	4 (4, 2)	171	0	159 (na)	150 (na)	163 (na)	157 (na)
G.D. Omar Torrijos Herrera National Park	Cloud	8°40”N /81° 35”W	262.2	25	3000	142	20 (na)	142	1 (1)	121 (na)	128 (1)	130 (1)	132 (1)
Montuosa Island Wildlife Refuge	Lowland Humid	7°28”N /82° 14”W	0.8	26	3500	206	14 (na)	206	0	200 (na)	199 (na)	202 (na)	200 (na)
Sarigua National Park	Lowland Dry	7°40”N /80° 35”W	46.6	27	1100	51	3 (na)	51	21 (11)	13 (5)	14 (11)	14 (5)	15 (5)
Soberania National Park	Lowland Humid	9°71”N /79° 42”W	195.4	28	2200	118	12 (na)	118	63 (35)	69 (21)	85 (28)	70 (21)	87
**Total Isolates (minimum number genotypes)**								**3307**	**1144 (291)**	**2121 (196)**	**2457 (245)**	**2474 (237)**	**2495 (244)**
**Total Highly Active Isolates (%)**										**358 (16.9)**	**378 (15.4)**	**99 (4.1)**	**140 (5.6)**

Because a high proportion of plants and fungi represent previously unknown or undescribed species, and not all collections or isolates were determined taxonomically, “minimum number” values refer to the subsets of plant and fungal samples that were identified to fine taxonomic levels.

Endophytic fungi were isolated from freshly collected, apparently healthy leaves following [37] with slight modifications. Within 48 h of collection, leaves were washed with tap water to remove excess debris. Twelve pieces measuring ca. 2 mm×2 mm were cut from each leaf, surface sterilized by sequential immersion in sodium hypochlorite (1%, 2 min) and ethanol (70%, 2 min) and then rinsed with sterile, distilled water. Leaf pieces were laid on the surface of 2% malt extract agar (MEA), a general medium that promotes growth by diverse endophytes [14] [32] [38], in 100 mm Petri dishes under sterile conditions. Dishes were sealed with Parafilm, stored at room temperature, and checked daily for three weeks for hyphal emergence. Emerging hyphae were cut from the plate under sterile conditions and transferred to axenic culture on 2% MEA. Isolates were stored as living vouchers at room temperature as agar plugs with mycelium in sterile distilled water and have been archived in the collection of the ICBG at the Smithsonian Tropical Research Institute in Panama (accessions available on request).

### Preparation of fungal extracts

A single plug was cut from actively growing, axenic mycelium, transferred aseptically to fresh 2% malt extract agar, and incubated at room temperature until mycelial growth covered at least 50% of the agar surface. Fifteen agar plugs (each 5 mm diameter) were cut with a sterile cork borer and transferred to flasks containing 37 ml of 2% malt extract broth. Flasks were incubated on an orbital shaker (28°C, 125 rpm) for one week. Those that were growing were left for seven additional days under the same conditions. Those that showed no visible signs of growth were removed from the orbital shaker and left at room temperature for three additional weeks to grow under static conditions.

Each liquid culture with evident growth was mixed with an equal volume of ethyl acetate (100%) and blended for 2 min at 9,000 rpm with a Polytron (Lauda-Brinkmann, Delran, NJ, USA). The resulting mixture was filtered with Whatman filter paper, #1 and transferred to a separation funnel where it was extracted twice with a 1∶1 volume of ethyl acetate. The aqueous phase was discarded and the organic layer was dried and stored at −80°C.

### Bioassays

Crude organic extracts of fungal cultures were used in bioassays against the causal agents of malaria (*Plasmodium falciparum*), leishmaniasis (*Leishmania donovani*) and Chagas' disease (*Trypanosoma cruzi*), and against the human breast cancer cell line MCF-7. Bioactivity of extracts, which were diluted in DMSO (10 μg/ml), was measured as percent inhibition of growth (% IG) compared to the negative control (DMSO with no extract; 0% IG). As a measure of susceptibility of target cells to known drugs, for each bioassay serial dilutions of the positive control were tested to determine IC50 values (i.e., half of the maximum inhibitory concentration). In some cases, % IG values obtained from bioassays exceeded 100% or were lower than 0% IG, consistent with enhancement of cell growth or destruction of existing cells relative to controls. All chemical reagents used for bioassays were obtained from Sigma Aldrich Inc. (Germany) unless specified.

The W2 strain of *Plasmodium falciparum*, obtained from the Malaria Research and Reference Reagent Resource Center (MR4, Manassas, VA, USA), was maintained in continuous culture following [39]. Cultures consisted of a 2% haematocrit suspension of O+ human erythrocytes in RPMI-1640 medium supplemented with a gentamicin solution (Gibco, Invitrogen, USA; 0.01 mg/mL), HEPES buffer (25 mM), NaHCO_3_ (AppliChem, USA; 25 mM), and human serum (10%). Cultures were supplied with a gas mixture consisting of 5% CO_2_, 5% O_2_, and 90% N_2_ and incubated in a cyclic incubator following [40]. Light microscopy with Giesma stain [41] was used to estimate parasitaemia and confirm parasite viability prior to bioassays. For each bioassay, 180 µl of culture and 20 µl of each extract was added to each well of a 96-well plate. The positive control consisted of chloroquine diluted in RPMI-1640 medium (normal IC50 value approximately 540 nM). After incubation for 48 h at 37°C, 50 µl of a PicoGreen cocktail (Invitrogen, USA) was added to each well. Fluorescence was determined at 485 nm in a plate reader (FL×800; BioTek Instruments Inc.) after 30 min.

The WR2801 strain of *Leishmania donovani donovani* (WR2801), a generous gift of Max Grogl (Experimental Therapeutics Division, Walter Reed Army Institute of Research, Silver Spring, MD, USA), was maintained as promastigotes in culture at 26°C in Schneider's medium amended with a 1∶2000 dilution of a penicillin-streptomycin mix (10,000 units of penicillin and 10 mg streptomycin/ml) and supplemented with sodium bicarbonate (4.6 mM) and 20% Fetal Bovine Serum (FBS; Cellgro, USA) at pH 7.2. Cells were transformed to amastigotes prior to bioassays by lowering the pH to 5.5 with HCl and incubating at 30°C for 4 days. For each biosassay, 1×10^6^ cells were placed in each well of a 96-well plate with 10 µl of extract in a final volume of 100 µl, and incubated for 3 days. The positive control was amphotericin B diluted in water (normal IC50 value 80–120 nM). A PicoGreen cocktail was added at a 1∶4 dilution and incubated at room temperature for 5 min before fluorescence was measured at 485 nm (as above).

The Tulahuen *LacZ* clone C4 of *Trypanosoma cruzi* parasites expressing β-galactosidase [42] was obtained from the American Type Culture Collection (ATCC, Manassas, VA, USA) and maintained in culture with RPMI-1640 supplemented with L-glutamine (Gibco; 200 mM), HEPES buffer (25 mM), NaHCO_3_ (25 mM), 1∶100 dilution of a penicillin-streptomycin mix (above) and FBS (10%) at 37°C. On the day prior to the bioassay, 1.2×10^4^ Vero cells (ATCC) were seeded in a final volume of 100 µl of culture medium/well in 96-well plates. After 24 h, Vero cells were infected with 5×10^4^ parasite cells, which had been diluted in 50 µl of culture medium. After an additional 24 h, 10 µg/ml of extract was added to each well and incubated at 37°C for 120 h. The positive control consisted of nifurtimox diluted in RPMI-1640 medium (normal IC50 value 0.5–1.5 μg/ml). Chlorophenol red-β-D-galactopyranoside colorimetric substrate (CPRG, Roche Applied Science) was then added and allowed to react with the β-galactosidase for 4.5 h at 37°C. Color intensity was read at 570 nm in a color plate reader (Sinergy HT, from BioTek Instruments Inc., Winooski, VT).

The MCF-7 mammalian breast cancer cell line was obtained from ATCC. On the day prior to the bioassay, 5×10^3^ cells were seeded in a final volume of 100 µl/well in 96-well plates and incubated with RPMI-640 supplemented with gentamicin (0.05 mg/ml), L-glutamine (GIBCO; 2 mM), NaHCO_3_ (4.6 mM), HEPES buffer (25 mM), and FBS (10%) at 37°C. For each bioassay, 100 µl of the extract was diluted in culture media, added to the cells, and incubated for 48 h at 37°C. Cells were fixed with tricholoroacetic acid (50%), rinsed with water, dried, and treated with 100 µl of sulphorhodamine B (0.4%), which was allowed to react for 15–30 min at 22°C. The positive control consisted of adriamycin diluted in DMSO (normal IC50 value 20–50 nM). The cells were then rinsed with trichloroacetic acid (1%), dried, and treated with Tris-HCl (10 mM; pH 7) for 15 min. Color intensity was read at 570 nm as described above.

### Identification of fungi

Total genomic DNA was extracted from fresh mycelium following [32]. The nuclear ribosomal internal transcribed spacers and 5.8 s gene (ITS rDNA) were amplified using primers ITS1F or ITS5 and ITS4 following [43]. PCR products were visualized using SYBR green following electrophoresis on a 1% agarose gel and positive amplicons were submitted to the University of Arizona Genetics Core for cleanup, normalization, and bidirectional Sanger sequencing. Sequences were assembled automatically and bases called using *phred* and *phrap* [44] [45] with orchestration by Mesquite [46], followed by manual editing in Sequencher 4.5 (GeneCodes Corp.). Edited consensus sequences were compared against the NCBI non-redundant database using BLASTn to estimate taxonomic placement at high taxonomic levels and submitted to GenBank under accession nos. KF435151-KF436419. Because identification based only on BLAST matches must be treated with caution [45] we used phylogenetic analyses following [21] to provide stronger inference regarding taxonomic affiliation. Genotype groups were determined by simultaneous comparison of edited consensus sequences for all strains in Sequencher 5.1 (Gene Codes Corp.) at 99% sequence similarity [32] [38] [47], which provides estimates of genotypic richness while allowing for a small amount of sequencing error [48].

### Statistical analysis

Statistical analyses were performed using JMP 10.0 (SAS Institute Inc., Cary, NC). Individual extracts and fungal genotypes were considered ‘highly active’ if they caused 50% or greater inhibition of growth (≥50% IG). When multiple isolates of the same genotype were examined, the genotype was considered highly active if the mean % IG of all isolates of that genotype or taxon was ≥50%. For analyses of variance based on host- and fungal taxonomy, we calculated the average bioactivity of endophytes belonging to each taxonomic group (e.g., family) and then assigned categorical variables as follows: low (<10% mean IG), moderate (10–20% mean IG), and high (>20% mean IG). Significant differences among categories were determined using Tukey's post-hoc test (CI = 95%).

## Results

Endophytes were isolated from 3198 plant collections representing 51 orders, 105 families and at least 232 genera of angiosperms and ferns collected in national parks throughout Panama. In sum, 3307 fungal endophytes were isolated; of these, ITS rDNA was sequenced for 1144 isolates (Table 1). Endophytes that were given taxonomic placement represented 291 genotypes belonging to 124 genera, 45 families and 21 orders. In total, 2723 isolates were tested for bioactivity; of these, 2118 were tested in all four assays, 571 isolates in three assays, 113 isolates in two assays, and 20 isolates in just one assay.

Bioactivity was observed among endophytes from diverse fungal lineages, host lineages, and collection sites. Overall, 32.7% of isolates demonstrated ≥50% IG (i.e., were highly active) in at least one bioassay. Of the 2118 isolates tested in all four bioassays, 0.6% were highly active in four assays, 1.7% in three assays, 5.4% in two assays, and 24.2% in only one assay. Approximately 4–17% of isolates tested in each assay were highly active, with the greatest frequency of highly active isolates observed in assays against *P. falciparum*, the causal agent of malaria (Table 1).

### Variation in bioactivity among fungal taxa

Analysis of all families and genera of fungi represented by at least three genotypes revealed (1) significant variation in mean % IG among fungal taxa in each bioassay and (2) that fungal lineages with the highest mean % IG differed between bioassays (Tables 2–9). Several fungal lineages were associated with little or no bioactivity in any of the four bioassays (Table 10).

**Table 2:** Activity of crude extracts from fungal endophytes against *Plasmodium falciparum* (causative agent of malaria) in *in vitro* assays, organized by fungal family.

Family (Order)	Mean %IG (± SE)	Fungal Genotypes Examined	Highly Active Genotypes	% Highly Active Genotypes	Activity Level
Mycosphaerellaceae (Capnodiales)	28.3 (6.8)	14	4	28.6	High
Trichocomaceae (Eurotiales)	26.7 (7.4)	12	3	25	High
Magnaporthaceae (Magnaporthales)	26.6 (11.5)	5	1	20	High
Xylariaceae (Xylariales)	20.6 (4)	41	5	12.2	High
Amphisphaeriaceae (Xylariales)	17.1 (9.7)	7	0	0	Moderate
Valsaceae (Diaporthales)	16.1 (5.3)	23	2	8.7	Moderate
Phyllachoraceae (Phyllachorales)	14.9 (6.4)	16	2	12.5	Moderate
Botryosphaeriaceae (Botryosphaeriales)	8.1 (11.5)	5	0	0	Low
Nectriaceae (Hypocreales)	6.7 (11.5)	5	0	0	Low

Data indicate mean percent inhibition of growth (mean % IG) of parasite cells and standard error, the number of fungal genotypes examined, the number and percent of those genotypes that are highly active (i.e., ≥50% IG), and a qualitative statement of activity level. The difference in mean % IG approached significance when the qualitative activity levels were compared (F_2,125_ = 2.57; p = 0.0804).

**Table 3:** Activity of crude extracts from fungal endophytes against *Plasmodium falciparum* (causative agent of malaria) in *in vitro* assays, organized by fungal genus.

Genus (Family)	Mean %IG (±SE)	Fungal Genotypes Examined	Highly Active Genotypes	% Highly Active Genotypes	Activity Level
*Glomerella* (Glomerellaceae)	50 (13)	5	3	60	High
*Daldinia* (Xylariaceae)	45.8 (14.5)	4	2	50	High
*Ophioceras* (Magnaporthaceae)	45.1 (16.8)	3	1	33.3	High
*Phomopsis* (Valsaceae)	35.6 (9.2)	10	3	30	High
*Mycosphaerella* (Mycosphaerellaceae)	31.9 (9.2)	10	4	40	High
*Aspergillus* (Trichocomaceae)	31.3 (14.5)	4	1	25	High
*Xylaria* (Xylariaceae)	26.4 (8.4)	12	3	25	High
*Diaporthe* (Diaporthaceae)	15.8 (13)	5	0	0	Moderate
*Colletotrichum* (Glomerellaceae)	12.7 (11)	7	0	0	Moderate
*Parapleurotheciopsis* (Xylariaceae)	11.1 (16.8)	3	0	0	Moderate
*Camarops* (Boliniaceae)	2.7 (16.8)	3	0	0	Low

Data indicate mean percent inhibition of growth of parasite cells (mean % IG) and standard error, the number of fungal genotypes examined, the number and percent of those genotypes that are highly active (i.e., ≥50% IG), and a qualitative statement of activity level. Mean % IG varied significantly among activity levels (F_2, 63_ = 4.78; p = 0.0117).

**Table 4:** Activity of crude extracts from fungal endophytes against *Leishmania donovani* (causative agent of leishmaniasis) in *in vitro* assays, organized by fungal family.

Family (Order)	Mean %IG (± SE)	Fungal Genotypes Examined	Highly Active Genotypes	% Highly Active Genotypes	Activity Level
Nectriaceae (Hypocreales)	34.9 (7.9)	7	2	28.6	High
Trichocomaceae (Eurotiales)	21.9 (5.2)	16	2	12.5	High
Mycosphaerellaceae (Capnodiales)	20.4 (5.2)	15	3	20	High
Amphisphaeriaceae (Xylariales)	18.8 (7.9)	7	0	0	Moderate
Xylariaceae (Xylariales)	17.7 (3.1)	46	6	13.1	Moderate
Valsaceae (Diaporthales)	15.8 (3.8)	31	1	3.2	Moderate
Botryosphaeriaceae (Botryosphaeriales)	15.5 (7.9)	7	0	0	Moderate
Phyllachoraceae (Phyllachorales)	9.5 (5.1)	17	0	0	Low
Boliniaceae (Boliniales)	7.9 (9.4)	5	0	0	Low
Magnaporthaceae (Magnaporthales)	3.9 (9.4)	5	0	0	Low

Data indicate mean percent inhibition of growth of parasite cells (mean % IG) and standard error, the number of fungal genotypes examined, the number and percent of those genotypes that are highly active (i.e., ≥50% IG), and a qualitative statement of activity level. Mean % IG varied significantly among activity levels (F_2, 153_ = 4.45; p = 0.0132).

**Table 5:** Activity of crude extracts from fungal endophytes against *Leishmania donovani* (causative agent of leishmaniasis) in *in vitro* assays, organized by fungal genus.

Genus (Family)	Mean %IG (± SE)	Fungal Genotypes Examined	Highly Active Genotypes	% Highly Active Genotypes	Activity Level
*Penidiella* (Incertae sedis)	42.3 (13.4)	3	1	33.3	High
*Diaporthe* (Diaporthaceae)	33.6 (10.4)	5	1	20	High
*Mycosphaerella* (Mycosphaerellaceae)	23.2 (8.2)	8	2	25	High
*Phomopsis* (Valsaceae)	21.6 (7.3)	10	2	20	High
*Aspergillus* (Trichocomaceae)	19 (10.4)	5	0	0	Moderate
*Xylaria* (Xylariaceae)	17.8 (6.2)	14	1	7.1	Moderate
*Daldinia* (Xylariaceae)	17 (10.4)	5	1	20	Moderate
*Colletotrichum* (Glomerellaceae)	10.5 (8.8)	7	0	0	Moderate
*Parapleurotheciopsis* (Xylariaceae)	7.2 (13.4)	3	0	0	Low
*Glomerella* (Glomerellaceae)	7.1 (9.5)	6	0	0	Low
*Ophioceras* (Magnaporthaceae)	6.4 (13.4)	3	0	0	Low
*Camarops* (Boliniaceae)	6.3 (11.6)	4	0	0	Low

Data indicate mean percent inhibition of growth of parasite cells (mean % IG) and standard error, the number of fungal genotypes examined, the number and percent of those genotypes that are highly active (i.e., ≥50% IG), and a qualitative statement of activity level. Mean % IG varied significantly among activity levels (F_2, 70_ = 4.21; p = 0.0188).

**Table 6:** Activity of crude extracts from fungal endophytes against *Trypanosoma cruzi* (causative agent of Chagas' disease) in *in vitro* assays, organized by fungal family.

Family (Order)	Mean %IG (± SE)	Fungal Genotypes Examined	Highly Active Genotypes	% Highly Active Genotypes	Activity Level
Nectriaceae (Hypocreales)	34.9 (3.6)	6	5	83.3	High
Trichocomaceae (Eurotiales)	23.5 (2.1)	16	3	18.8	High
Valsaceae (Diaporthales)	22.1 (1.6)	33	6	18.2	High
Mycosphaerellaceae (Capnodiales)	18.1 (2.6)	16	2	12.5	Moderate
Amphisphaeriaceae (Xylariales)	13.6 (3.5)	7	0	0	Moderate
Phyllachoraceae (Phyllachorales)	13.5 (1.3)	17	2	11.8	Moderate
Magnaporthaceae (Magnaporthales)	13.1 (4.1)	5	0	0	Moderate
Xylariaceae (Xylariales)	12.8 (1.3)	45	2	4.4	Moderate
Botryosphaeriaceae (Botryosphaeriales)	12.4 (2.9)	8	0	0	Moderate

Data indicate mean percent inhibition of growth of parasite cells (mean % IG) and standard error, the number of fungal genotypes examined, the number and percent of those genotypes that are highly active (i.e., ≥50% IG), and a qualitative statement of activity level. Mean % IG varied significantly among activity levels (F_1,510_ = 53.2; p = <0.0001).

**Table 7:** Activity of crude extracts from fungal endophytes against *Trypanosoma cruzi* (causative agent of Chagas' disease) in *in vitro* assays, organized by fungal genus.

Genus (Family)	Mean %IG (± SE)	Fungal Genotypes Examined	Highly Active Genotypes	% Highly Active Genotypes	Activity Level
*Phomopsis* (Valsaceae)	30.4 (4.1)	11	2	18.2	High
*Diaporthe* (Diaporthaceae)	23.6 (5.5)	6	1	16.7	High
*Aspergillus* (Trichocomaceae)	18.9 (6)	5	0	0	High
*Penidiella* (Incertae sedis)	17.8 (7.8)	3	0	0	Moderate
*Colletotrichum* (Glomerellaceae)	16.1 (5.1)	7	0	0	Moderate
*Mycosphaerella* (Mycosphaerellaceae)	16 (4.1)	11	0	0	Moderate
*Xylaria* (Xylariaceae)	15 (3.6)	14	1	7.1	Moderate
*Glomerella* (Glomerellaceae)	13.7 (6)	5	0	0	Moderate
*Daldinia* (Xylariaceae)	11.8 (6.8)	4	0	0	Low
*Camarops* (Boliniaceae)	11.2 (7.8)	3	0	0	Low
*Ophioceras* (Magnaporthaceae)	10.8 (7.8)	3	0	0	Low

Data indicate mean percent inhibition of growth of parasite cells (mean % IG) and standard error, the number of fungal genotypes examined, the number and percent of those genotypes that are highly active (i.e., ≥50% IG), and a qualitative statement of activity level. Mean % IG varied significantly among activity levels (F_1,181_ = 4.16; p = 0.0428).

**Table 8:** Activity of crude extracts from fungal endophytes against MCF-7 breast cancer cell line in *in vitro* assays, organized by fungal family.

Family (Order)	Mean %IG (± SE)	Fungal Genotypes Examined	Highly Active Genotypes	% Highly Active Genotypes	Activity Level
Nectriaceae (Hypocreales)	38.7 (8.1)	6	2	33.3	High
Trichocomaceae (Eurotiales)	24 (4.8)	17	4	23.5	High
Valsaceae (Diaporthales)	15.7 (3.5)	32	3	9.4	Moderate
Xylariaceae (Xylariales)	13.3 (2.9)	45	3	6.7	Moderate
Amphisphaeriaceae (Xylariales)	8 (7.5)	7	0	0	Low
Magnaporthaceae (Magnaporthales)	7.7 (8.9)	5	0	0	Low
Phyllachoraceae (Phyllachorales)	6 (4.7)	17	0	0	Low
Botryosphaeriaceae (Botryosphaeriales)	5 (6.6)	9	0	0	Low
Boliniaceae (Boliniales)	4.6 (8.9)	5	0	0	Low
Mycosphaerellaceae (Capnodiales)	3.9 (4.8)	17	0	0	Low

Data indicate mean percent inhibition of growth of parasite cells (mean % IG) and standard error, the number of fungal genotypes examined, the number and percent of those genotypes that are highly active (i.e., ≥50% IG), and a qualitative statement of activity level. Mean % IG varied significantly among activity levels (F_2, 160_ = 11.12; p = <0.0001).

**Table 9:** Activity of crude extracts from fungal endophytes against MCF-7 breast cancer cell line in *in vitro* assays, organized by fungal genus.

Genus (Family)	Mean %IG (± SE)	Fungal Genotypes Examined	Highly Active Genotypes	% Highly Active Genotypes	Activity Level
*Aspergillus* (Trichocomaceae)	41.6 (7.5)	5	3	60	High
*Xylaria* (Xylariaceae)	20.2 (4.5)	14	1	7.1	High
*Phomopsis* (Valsaceae)	18.8 (5)	11	0	0	Moderate
*Daldinia* (Xylariaceae)	10.2 (7.5)	5	0	0	Moderate
*Guignardia* (Botryosphaeriaceae)	8.4 (9.6)	3	0	0	Low
*Ophioceras* (Magnaporthaceae)	8.4 (9.6)	3	0	0	Low
*Mycosphaerella* (Mycosphaerellaceae)	6.3 (5)	11	0	0	Low
*Camarops* (Boliniaceae)	5.5 (8.4)	4	0	0	Low
*Colletotrichum* (Glomerellaceae)	4 (6.3)	7	0	0	Low
*Glomerella* (Glomerellaceae)	3.5 (6.8)	6	0	0	Low
*Diaporthe* (Diaporthaceae)	3.4 (7.5)	5	0	20	Low
*Phoma* (Incertae sedis)	2.6 (9.6)	3	0	0	Low
*Parapleurotheciopsis* (Xylariaceae)	1.6 (9.6)	3	0	0	Low

Data indicate mean percent inhibition of growth of parasite cells (mean % IG) and standard error, the number of fungal genotypes examined, the number and percent of those genotypes that are highly active (i.e., ≥50% IG), and a qualitative statement of activity level. Mean % IG varied significantly among activity levels (F_2,78_ = 7.18; p = 0.0014).

**Table 10:** Summary of fungal and host plant lineages with endophytes that demonstrated especially low mean % inhibition of growth across multiple bioassays, and few or no highly bioactive genotypes.

Taxonomic group	Malaria		Leishmaniasis		Chagas' Disease		Cancer	
	Mean % IG	% Highly Active Genotypes	Mean % IG	% Highly Active Genotypes	Mean % IG	% Highly Active Genotypes	Mean % IG	% Highly Active Genotypes
*Fungal Family (Order)*								
Amphisphaeriaceae (Xylariales)	17.1	0	18.8	0	13.6	0	8	0
Boliniaceae (Boliniales)	na	na	7.9	0	na	na	4.6	0
Botryosphaeriaceae (Botryosphaeriales)	8.1	0	15.5	0	12.4	0	5	0
Phyllachoraceae (Phyllachorales)	14.9	12.5	9.5	0	13.5	11.8	6	0
*Host Plant Family (Order)*								
Euphorbiaceae (Malpighiales)	21.5	16.7	10.2	0	16.8	0	3	0
Annonaceae (Magnoliales)	13.7	11.1	4.9	0	10.9	0	6.3	0

Taxonomic groups for which endophytes were not tested in a given assay are marked ‘na’.

### Variation in bioactivity at the level of host plant taxa

We evaluated the mean % IG of endophytes from all plant families from which at least three endophyte genotypes were isolated. Mean % IG differed significantly among endophytes representing various plant families. Host plant families associated with endophytes that displayed the highest mean % IG also differed among bioassays (Tables S1 – S4 in File S1 ). Several plant families harbored endophytes that had little or no bioactivity in the four bioassays (Table 10).

### Differences in bioactivity as a function of forest type

**Figure 1:**
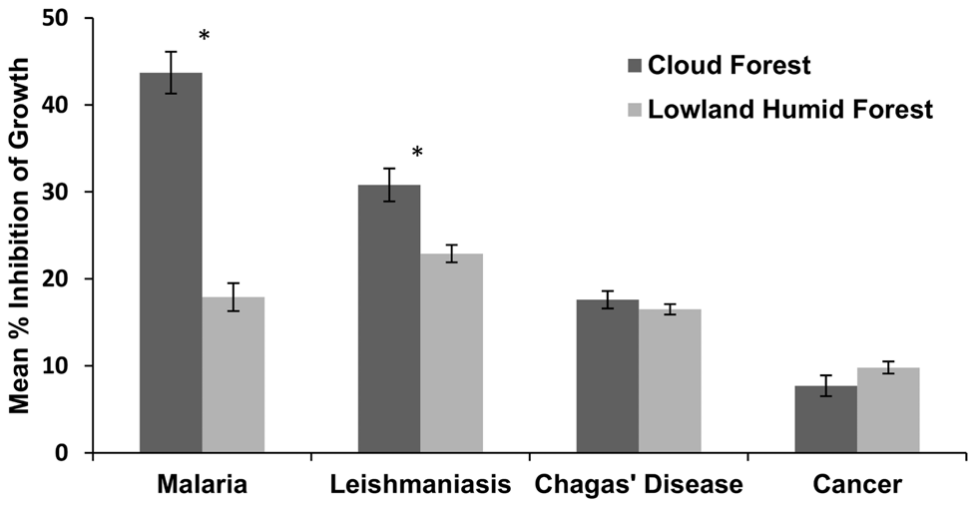
Mean % IG by forest type. Mean % inhibition of growth of the causative agents of malaria, leishmaniasis and Chagas' disease, and against the MCF-7 breast cancer cell line, as a function of forest type. The analyses included fungi from all host plant orders with at least three isolates in each of the two forest types. Asterisks denote significant differences within a given assay.

Because the majority of our sampling was conducted in cloud forest and lowland humid forest (Table 1), we focused on those two forest types, and only considered endophytes represented by at least three isolates from each plant taxa. We found that cloud forest endophytes had significantly higher mean % IG than lowland humid forest endophytes against both *P. falciparum* (F_29, 665_ = 8.54, p = 0.0001) and *L. donovani* (F_16,931_ = 2.07, p = 0.0079) *in vitro*. No meaningful differences in mean % IG were observed against *T. cruzi* or MCF-7 breast cancer cells *in vitro* (Figure 1).

To distinguish whether these significant differences reflected the presence of different major lineages of fungi in cloud vs. lowland forest, or different levels of activity among the same major lineages in these forests, we examined the most prevalent fungal orders that were identified in both forest types, and for which sufficient bioassay results were available. Three orders fit these criteria: Capnodiales, Dothideales, and Phyllachorales. In each case, we observed a trend for higher activity, or significantly higher activity, in at least one bioassay by cloud forest strains relative to strains belonging to the identical order from lowland humid forests (data not shown). We further examined the bioactivity of the most common fungal genotypes that occurred in both cloud forests and lowland humid forests. Analyses were restricted to genotypes that were represented by at least 10 isolates, were obtained from both forest types, and were assessed for bioactivity in at least two assays. Three genotypes fit these criteria. In all three, strains from cloud forests demonstrated significantly greater bioactivity than conspecifics from lowland forest in at least one bioassay (Z-tests for exact means; alpha  = 0.05; p<0.05 in all cases).

## Discussion

The enormous cost of drug development is a clear incentive for pharmaceutical companies to disregard all but the most financially viable lead compounds. However, the dwindling number of medications reaching the market is putting intense pressure on the industry to innovate [6]. Combinatorial chemistry, coupled with the new high-throughput screening (HTS) technology of the early 1990's, seemed to promise a new era of drug discovery success [49]. However, from 1981 to 2010 only one *de novo* combinatorial compound was approved by the FDA [50]. Meanwhile, natural products and natural product structures, in particular those from microbial sources, continue to be reported in considerable numbers [51]. Many microorganisms appear to be intrinsically capable of producing far more natural products than have been observed in the lab [52], thus representing a rich source of novel, bioactive metabolites. Tropical fungal endophytes are of particular interest as they exhibit remarkable abundance and diversity, and communities differ markedly at regional and large geographic scales [32] [37]. Furthermore, endophytes are thought to use chemical compounds to mediate interactions with competitors and other antagonists [53] [54]. Our collections across a diversity of biomes in Panama suggest taxonomic and ecological attributes that might enhance our ability to discover bioactive compounds for particular disease targets.

Our analyses show for the first time that endophytes isolated from plants in cloud forests are considerably more bioactive in assays against *P. falciparum* and *L. donovani* than those isolated from plants in lowland humid forests (Figure 1). Our results suggest that even when the same fungal taxa are found in lowland forests, those isolated from cloud forests demonstrate greater bioactivity. We hypothesize that the moist conditions of cloud forests may enhance the colonization of leaves by endophytes (as for epiphylls; [55]): the frequency with which endophytes are isolated from tissues tends to be negatively associated with desiccation and ultraviolet radiation [56] [57], and positively associated with leaf lifetime and humidity [14] [54]. The seasonal lowland forests we examined have greater seasonal drought stress and UV irradiance, and a higher proportion of seasonally deciduous species, than the cloud forests we considered here. Future studies linking bioactivity *in vitro* with bioactivity in symbiosis may be especially illuminating with regard to efficient recovery of bioactive endophytes.

Our analyses also highlight taxonomic groups of plants that harbored (1) a high percentage of highly active endophytes, and (2) endophytes with high mean % IG in several bioassays (Tables S1 – S4 in File S1). For example, fungi isolated from the plant family Araceae (Alismatales) had a high percentage of highly active genotypes and were associated with moderate to high mean % IG against *L. donovani, T. cruzi* and the MCF-7 breast cancer cell line. Fungi isolated from the plant family Fabaceae (Fabales) had a high percentage of highly active genotypes and were associated with moderate to high mean % IG against *P. falciparum* and the MCF-7 breast cancer cell line.

The bioactivity profiles of several fungal lineages suggested compounds with strong and specific bioactivity (Tables 2–9). Specific bioactivity, defined as high inhibition of growth of one type of target organism with little or no activity against others, is of particular interest in drug discovery: it suggests the presence of compounds that have specific modes of action as opposed to highly toxic compounds that are often of little use as medication [58]. Extracts from fungi of the family Mycosphaerellaceae (Capnodiales) had moderate to high mean % IG against the parasites *P. falciparum, L. donovani* and *T. cruzi*, but had particularly low mean % IG against the MCF-7 breast cancer cell line. In contrast, under the conditions used here, extracts from fungi of the family Trichocomaceae (Eurotiales) had high mean % IG against the three tropical disease parasites as well as against the MCF-7 breast cancer cell line, suggesting the presence of non-specific and highly toxic compounds. As such, fungi from this family might be excluded from future scale-up efforts for greatest efficiency.

Tropical fungal endophytes are a well-documented source of interesting bioactive metabolites and, with their immense biodiversity, hold enormous potential for future drug discovery [59]. Here, we used only a single isolation medium and isolation approach to obtain endophytes from photosynthetic tissues; thus it is likely that additional genotypes with specialized growth requirements, or that inhabit other tissues, would be discovered through further research. Similarly, additional bioactivity might be observed under different growth conditions, as secondary metabolite production is strongly influenced by factors such as substrate type, temperature, and other factors [60]. However, our focus on foliar endophytes and the methods outlined here has been fruitful: the Panama ICBG has had much success discovering novel bioactive compounds from these organisms (e.g., [61] [62] [63]).

Overall, more than 32% of our fungal isolates were active in at least one of the four bioassays. However, by analyzing >10 years of collection and bioassay data we have shown that by tailoring certain selection criteria we could significantly improve our chances of encountering highly bioactive fungi. For example, we observed that 16.9% of fungal genotypes screened in our work were highly active against *P. falciparum*. If only those fungi isolated from host plants of the family Fabaceae (Fabales) are considered, the prevalence of highly active isolates increases to 22.2%. If only host plants of the order Fabales that were collected from cloud forests are considered, the prevalence of highly active isolates increases to 53.1% – a more than three-fold improvement that could dramatically increase the chances of finding interesting bioactive molecules while maximizing limited resources. Analyses of this type, although not yet vetted for predictive power in other forests, are important for establishing guidelines to enhance the efficacy and efficiency of future bioprospecting efforts.

## Supporting Information

File S1 **Supporting Information file that contains. Table S1. Activity of fungal endophytes against** ***Plasmodium falciparum*** **(causative agent of malaria) in** ***in vitro*** **assays, organized by host plant family.** Data indicate mean percent inhibition of growth (mean % IG) of parasite cells and standard error, the number of plant species and fungal genotypes examined, the number and percent of fungal genotypes that are highly active (i.e. ≥50% IG), and a qualitative statement of activity level. Mean % IG varied significantly between activity levels (F_2,137_ = 4.67; p = 0.0108). **Table S2. Activity of fungal endophytes against** ***Leishmania donovani*** **(causative agent of leishmaniasis) in** ***in vitro*** **assays, organized by host plant family.** Data indicate mean percent inhibition of growth (mean % IG) of parasite cells and standard error, the number of plant species and fungal genotypes examined, the number and percent of fungal genotypes that are highly active (i.e. ≥50% IG), and a qualitative statement of activity level. Mean % IG varied significantly between activity levels (F_2,194_ = 4.86; p = 0.0087). **Table S3. Activity of fungal endophytes against** ***Trypanosoma cruzi*** **(causative agent of Chagas' disease) in** ***in vitro*** **assays, organized by host plant family.** Data indicate mean percent inhibition of growth (mean % IG) of parasite cells and standard error, the number of plant species and fungal genotypes examined, the number and percent of fungal genotypes that are highly active (i.e. ≥50% IG), and a qualitative statement of activity level. Mean % IG varied significantly between activity levels (F_1,181_ = 4.20; p = 0.0428). **Table S4. Activity of fungal endophytes against MCF-7 breast cancer cells in** ***in vitro*** **assays, organized by host plant family.** Data indicate mean percent inhibition of growth (mean % IG) of parasite cells and standard error, the number of plant species and fungal genotypes examined, the number and percent of fungal genotypes that are highly active (i.e. ≥50% IG), and a qualitative statement of activity level. Mean % IG varied significantly between activity levels (F_2,171_ = 4.49; p = 0.0125).
(DOC)
