# Supplementary material for: Bioactivity of Fungal Endophytes as a Function of Endophyte Taxonomy and the Taxonomy and Distribution of Their Host Plants
Source: PLoS One. 2013 Sep 16;8(9):e73192. doi: 10.1371/journal.pone.0073192 (PMC3774686; doi:10.1371/journal.pone.0073192)
Supplement: File S1 — Supporting Information file that contains. Table S1. Activity of fungal endophytes against Plasmodium falciparum (causative agent of malaria) in in vitro assays, organized by host plant family. Data indicate mean percent inhibition of growth (mean % IG) of parasite cells and standard error, the number of plant species and fungal genotypes examined, the number and percent of fungal genotypes that are highly active (i.e. ≥50% IG), and a qualitative statement of activity level. Mean % IG varied significantly between activity levels (F2,137 = 4.67; p = 0.0108). Table S2. Activity of fungal endophytes against Leishmania donovani (causative agent of leishmaniasis) in in vitro assays, organized by host plant family. Data indicate mean percent inhibition of growth (mean % IG) of parasite cells and standard error, the number of plant species and fungal genotypes examined, the number and percent of fungal genotypes that are highly active (i.e. ≥50% IG), and a qualitative statement of activity level. Mean % IG varied significantly between activity levels (F2,194 = 4.86; p = 0.0087). Table S3. Activity of fungal endophytes against Trypanosoma cruzi (causative agent of Chagas' disease) in in vitro assays, organized by host plant family. Data indicate mean percent inhibition of growth (mean % IG) of parasite cells and standard error, the number of plant species and fungal genotypes examined, the number and percent of fungal genotypes that are highly active (i.e. ≥50% IG), and a qualitative statement of activity level. Mean % IG varied significantly between activity levels (F1,181 = 4.20; p = 0.0428). Table S4. Activity of fungal endophytes against MCF-7 breast cancer cells in in vitro assays, organized by host plant family. Data indicate mean percent inhibition of growth (mean % IG) of parasite cells and standard error, the number of plant species and fungal genotypes examined, the number and percent of fungal genotypes that are highly active (i.e. ≥50% IG), and a qualitative sta [file pone.0073192.s001.doc]

**Table S1**: Activity of fungal endophytes against *Plasmodium falciparum* (causative agent of malaria) in *in vitro* assays, organized by host plant family. Data indicate mean percent inhibition of growth (mean % IG) of parasite cells and standard error, the number of plant species and fungal genotypes examined, the number and percent of fungal genotypes that are highly active (i.e. ≥ 50 % IG), and a qualitative statement of activity level. Mean % IG varied significantly between activity levels (F_2,137_ = 4.67; p = 0.0108).

| **Family (Order)** | **Mean %IG (± SE)** | **Plant Species Examined** | **Fungal Genotypes Examined** | **Highly Active Genotypes** | **% Highly Active Genotypes** | **Activity Level** |
| --- | --- | --- | --- | --- | --- | --- |
| Bignoniaceae (Scrophulariales) | 51.1 (11.4) | 3 | 6 | 3 | 50 | High |
| Clusiaceae (Malpighiales) | 38.5 (14) | 3 | 4 | 2 | 50 | High |
| Fabaceae (Fabales) | 25.5 (4.2) | 13 | 36 | 10 | 27.8 | High |
| Apocynaceae (Gentianales) | 19.7 (7) | 3 | 14 | 2 | 14.3 | Moderate |
| Rubiaceae (Gentianales) | 17.8 (7) | 10 | 14 | 2 | 14.3 | Moderate |
| Euphorbiaceae (Malpighiales) | 16.1 (9.9) | 4 | 8 | 1 | 12.5 | Moderate |
| Verbenaceae (Lamiales) | 15.9 (7.8) | 4 | 13 | 1 | 7.7 | Moderate |
| Annonaceae (Magnoliales) | 12.4 (8.9) | 3 | 9 | 1 | 11.1 | Moderate |
| Araceae (Alismatales) | 10.6 (10.6) | 3 | 7 | 1 | 14.3 | Moderate |
| Moraceae (Rosales) | 9.6 (7.2) | 4 | 14 | 0 | 0 | Low |

**Table S2**: Activity of fungal endophytes against *Leishmania donovani* (causative agent of leishmaniasis) in *in vitro* assays, organized by host plant family. Data indicate mean percent inhibition of growth (mean % IG) of parasite cells and standard error, the number of plant species and fungal genotypes examined, the number and percent of fungal genotypes that are highly active (i.e. ≥ 50 % IG), and a qualitative statement of activity level. Mean % IG varied significantly between activity levels (F_2,194_ = 4.86; p = 0.0087).

| **Family (Order)** | **Mean %IG (± SE)** | **Plant Species Examined** | **Fungal Genotypes Examined** | **Highly Active Genotypes** | **% Highly Active Genotypes** | **Activity Level** |
| --- | --- | --- | --- | --- | --- | --- |
| Bignoniaceae (Scrophulariales) | 24.8 (9.2) | 3 | 6 | 0 | 0 | High |
| Rubiaceae (Gentianales) | 21.9 (4.1) | 10 | 25 | 2 | 8 | High |
| Apocynaceae (Gentianales) | 20 (5.3) | 3 | 17 | 2 | 11.8 | High |
| Verbenaceae (Lamiales) | 17.6 (4.7) | 5 | 22 | 3 | 13.6 | Moderate |
| Araceae (Alismatales) | 15.8 (7.5) | 4 | 9 | 1 | 11.1 | Moderate |
| Moraceae (Rosales) | 15.1 (4.6) | 5 | 20 | 2 | 10 | Moderate |
| Fabaceae (Fabales) | 14.9 (3.2) | 15 | 41 | 4 | 9.8 | Moderate |
| Euphorbiaceae (Malpighiales) | 9.4 (6.2) | 3 | 12 | 0 | 0 | Low |
| Clusiaceae (Malpighiales) | 6.2 (6) | 3 | 13 | 0 | 0 | Low |
| Annonaceae (Magnoliales) | 4.4 (7.1) | 3 | 9 | 0 | 0 | Low |

**Table S3**: Activity of fungal endophytes against *Trypanosoma cruzi* (causative agent of Chagas’ disease) in *in vitro* assays, organized by host plant family. Data indicate mean percent inhibition of growth (mean % IG) of parasite cells and standard error, the number of plant species and fungal genotypes examined, the number and percent of fungal genotypes that are highly active (i.e. ≥ 50 % IG), and a qualitative statement of activity level. Mean % IG varied significantly between activity levels (F_1,181_ = 4.20; p = 0.0428).

| **Family (Order)** | **Mean %IG (± SE)** | **Plant Species Examined** | **Fungal Genotypes Examined** | **Highly Active Genotypes** | **% Highly Active Genotypes** | **Activity Level** |
| --- | --- | --- | --- | --- | --- | --- |
| Fabaceae (Fabales) | 20.8 (2.3) | 15 | 39 | 6 | 15.4 | High |
| Rubiaceae (Gentianales) | 20.8 (3.1) | 10 | 23 | 3 | 13.1 | High |
| Bignoniaceae (Scrophulariales) | 20.6 (6.7) | 3 | 6 | 0 | 0 | High |
| Araceae (Alismatales) | 19.8 (5.5) | 4 | 9 | 1 | 11.1 | Moderate |
| Apocynaceae (Gentianales) | 17.8 (3.9) | 3 | 17 | 1 | 5.9 | Moderate |
| Euphorbiaceae (Malpighiales) | 16.8 (4.9) | 3 | 11 | 0 | 0 | Moderate |
| Clusiaceae (Malpighiales) | 15.5 (4.7) | 3 | 12 | 0 | 0 | Moderate |
| Verbenaceae (Lamiales) | 15.3 (3.3) | 5 | 22 | 1 | 4.5 | Moderate |
| Moraceae (Rosales) | 15.1 (3.8) | 5 | 16 | 1 | 6.3 | Moderate |
| Annonaceae (Magnoliales) | 10.9 (5.8) | 3 | 8 | 0 | 0 | Moderate |

**Table S4**: Activity of fungal endophytes against MCF-7 breast cancer cells in *in vitro* assays, organized by host plant family. Data indicate mean percent inhibition of growth (mean % IG) of parasite cells and standard error, the number of plant species and fungal genotypes examined, the number and percent of fungal genotypes that are highly active (i.e. ≥ 50 % IG), and a qualitative statement of activity level. Mean % IG varied significantly between activity levels (F_2,171_ = 4.49; p = 0.0125).

| **Family (Order)** | **Mean %IG (± SE)** | **Plant Species Examined** | **Fungal Genotypes Examined** | **Highly Active Genotypes** | **% Highly Active Genotypes** | **Activity Level** |
| --- | --- | --- | --- | --- | --- | --- |
| Araceae (Alismatales) | 27.6 (10.7) | 3 | 5 | 1 | 20 | High |
| Fabaceae (Fabales) | 16.5 (3.7) | 11 | 36 | 6 | 16.7 | Moderate |
| Rubiaceae (Gentianales) | 16.3 (4.5) | 10 | 23 | 4 | 17.4 | Moderate |
| Moraceae (Rosales) | 13.6 (4.9) | 5 | 20 | 2 | 10 | Moderate |
| Apocynaceae (Gentianales) | 10.9 (6) | 3 | 16 | 1 | 6.3 | Moderate |
| Bignoniaceae (Scrophulariales) | 10.8 (11.9) | 3 | 4 | 0 | 0 | Moderate |
| Verbenaceae (Lamiales) | 6.3 (5.2) | 5 | 20 | 0 | 0 | Low |
| Annonaceae (Magnoliales) | 6.3 (9) | 3 | 7 | 0 | 0 | Low |
| Clusiaceae (Malpighiales) | 3.8 (6.2) | 4 | 13 | 0 | 0 | Low |
| Euphorbiaceae (Malpighiales) | 3 (6.9) | 3 | 12 | 0 | 0 | Low |
